# Associations of a prognostic immune and nutritional index with all-cause and cause-specific mortality in individuals with depression: evidence from NHANES 2005–2018

**DOI:** 10.3389/fnut.2025.1588896

**Published:** 2025-07-11

**Authors:** Wendong Fang, Jie Zhang, Wanxian Gong, Yuanhong Xu, Lei Sun

**Affiliations:** ^1^Department of Clinical Laboratory, The Lu'an People's Hospital of Anhui Medical University, Lu'an, Anhui, China; ^2^Department of Clinical Laboratory, The First Affiliated Hospital of Anhui Medical University, Hefei, Anhui, China

**Keywords:** NHANES, prognostic immune and nutritional index, PINI, depression, mortality

## Abstract

**Background:**

The prognostic immune and nutritional index (PINI) has been used to predict survival outcomes in colon cancer patients. However, the relationship between the PINI and survival outcomes in patients with depression remains unclear. This study aimed to explore the association of the PINI with all-cause and cause-specific mortality in patients with depression.

**Methods:**

This study enrolled 6,610 patients with depression from the National Health and Nutrition Examination Survey (2005–2018). Mortality outcomes were determined by National Death Index records on 31 December 2019. Weighted Cox proportional hazards models were used to estimate hazard ratios (HRs) and 95% confidence intervals (95% CIs) for all-cause and cause-specific mortality. Kaplan–Meier curves were used to visualize survival probabilities at PINI levels, and subgroup analyses were performed to assess interactions with key variables. Smoothed curve fitting was also used to examine the non-linear relationship between the PINI and various mortality outcomes in the association.

**Results:**

During a median follow-up of 6.5 years, a total of 702 all-cause deaths and 178 cardiovascular disease (CVD) deaths were recorded. Higher PINI quartiles were associated with lower all-cause, CVD, and cancer mortality (all *P* <0.001). Cox regression showed that the highest PINI quartile had significantly lower hazard ratios for all-cause mortality (HR = 0.458, 95% CI: 0.349–0.603), CVD mortality (HR = 0.258, 95% CI: 0.134–0.498), and cancer mortality (HR = 0.554, 95% CI: 0.284–1.081). A smooth curve fitting analysis revealed an L-shaped inverse relationship between the PINI and all-cause, CVD, and cancer mortality.

**Conclusion:**

Higher PINI levels are associated with significantly lower all-cause and CVD mortality in individuals with depression. More large-scale and diverse population studies are needed to clarify the effects of higher PINI levels on all-cause and specific mortality.

## Introduction

Depression is a prevalent and debilitating mental disorder affecting millions worldwide, with significant effects on both individual wellbeing and public health (1, 2). According to the World Health Organization, depression contributes to the overall global burden of disease and is associated with high morbidity and mortality rates (3–5). Despite numerous advances in psychiatric treatment, the prognosis for individuals with depression varies considerably due to its complex etiology involving genetic, psychological, and environmental factors (6–8).

Recent research has increasingly focused on the effects of physical health on mental disorders, particularly the interplay between nutritional status and immune function in depression (9, 10). Nutritional deficiencies and immune dysregulation are common in individuals with depression and are thought to influence depression severity and progression (9, 11). However, despite considerable investigation into these associations, the combined impact of immune and nutritional status—encapsulated by the prognostic immune and nutritional index (PINI)—remains underexplored in individuals with depression. The PINI has been used in certain clinical areas, including oncology and surgery, to predict outcomes and tailor interventions (12, 13).

The PINI has been shown to predict outcomes in the colon cancer population, but its role in other populations, particularly those with depression, is unclear. This study aimed to address this gap. Using National Health and Nutrition Examination Survey (NHANES) 2005–2018 data, we examined the link between the PINI and mortality in depressed individuals.

## Materials and methods

### Study design and participants

NHANES uses a sophisticated and complex multistage probability sampling design to select a representative sample of the U.S. population every 2 years. The primary objective of the NHANES is to assess the health and nutritional status of adults and children in the United States through a combination of interviews, physical examinations, and laboratory tests. The survey protocols were approved by the National Center for Health Statistics (NCHS) Institutional Review Board, and informed written consent was obtained from all participants. We adhered to the Strengthening the Reporting of Observational Studies in Epidemiology (STROBE) guidelines for reporting observational studies to ensure transparency and quality in our analysis of the NHANES data.

**Figure 1:**
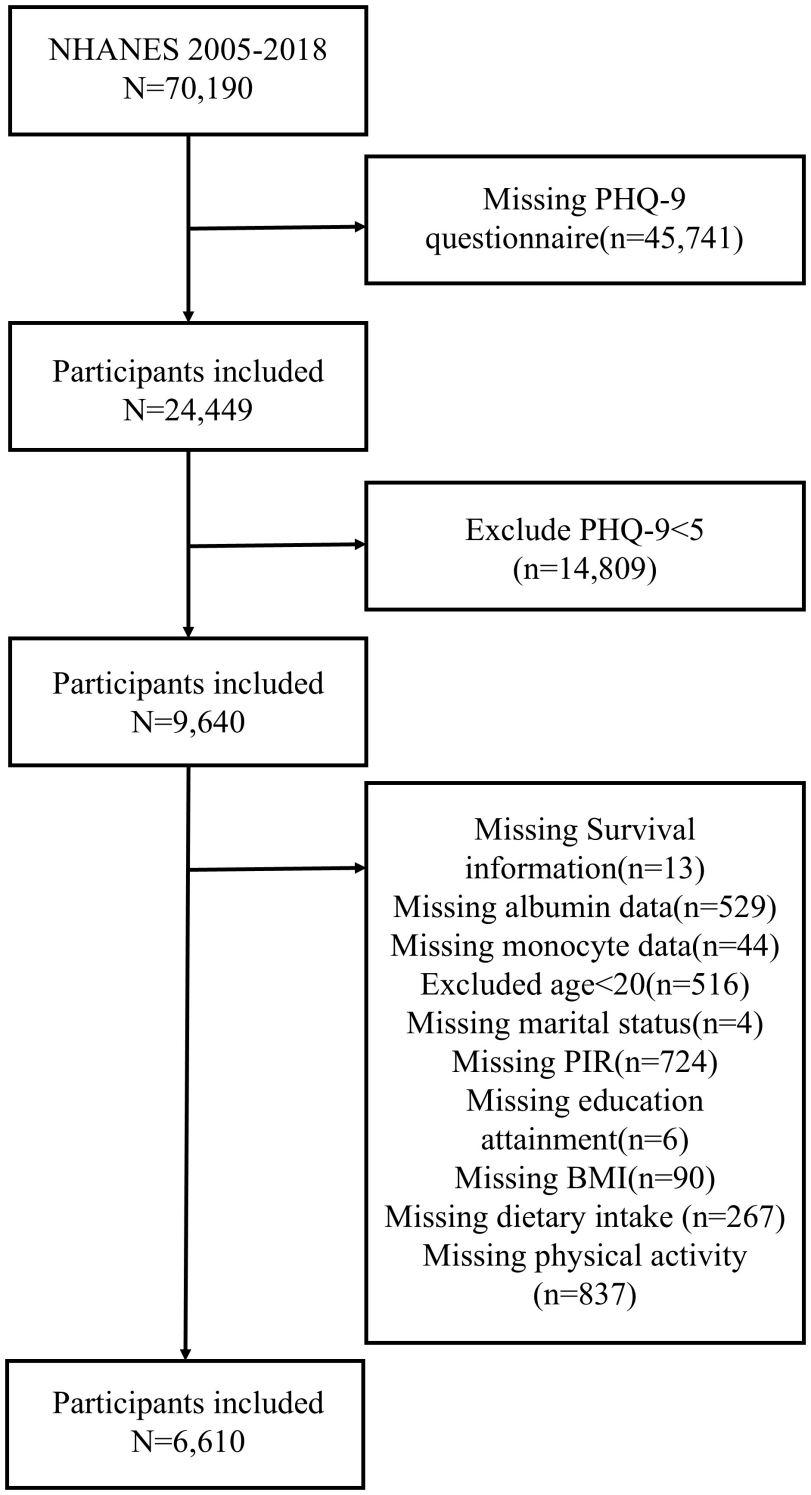
Flowchart of participant inclusion and exclusion.

To support the reliability and transparency of the results, the filter criteria were recorded in detail. In this study, we analyzed data from the NHANES 2005 to 2018 cycles (*n* = 70,190). First, 45,741 participants who did not complete the Patient Health Questionnaire-9 (PHQ-9) were excluded. Second, according to the diagnostic criteria of the PHQ-9, 14,809 individuals without depressive tendencies were excluded (PHQ-9 score <5 was considered no tendency for depression) (14, 15). Individuals with missing covariate data were removed, including those missing albumin data (*n* = 529), monocyte data (*n* = 44), survival information (*n* = 13), marital status data (*n* = 4), PIR data (*n* = 724), education attainment (*n* = 6), body mass index (BMI) data (*n* = 90), dietary intake data (*n* = 267), and physical activity data (*n* = 837). Individuals <20 years of age were also excluded (*n* = 516). After applying these exclusion criteria, a total of 6,610 participants with depression were included in the study. The detailed inclusion and exclusion processes are illustrated in Figure 1.

### Exposure and outcome variables

The primary exposure variable in this study was the PINI, which was calculated using the formula:


$$PINI=[\text{albumin }(\text{g/dL})\times \text{0}.\text{9 − }[\text{monocytes }({\text{mm}}^{\text{3}})\times \text{0}.\text{0007}]]$$

The PINI is an established index that reflects the balance between the nutritional and immune status, with higher values indicating a better prognosis (12, 13, 16).

The primary outcome of interest was all-cause mortality, with additional analyses conducted for cause-specific mortality. Mortality status and cause of death were ascertained using the NHANES public-use linked mortality file, updated on 31 December 2019. This file was linked to the National Death Index by the NCHS using a probability matching algorithm to ensure accurate identification of mortality outcomes.

Cause-specific mortality was categorized according to the International Statistical Classification of Diseases, 10th Revision (ICD-10). In this study, we focused on deaths due to heart diseases (ICD-10 codes 054–064), malignant neoplasms (ICD-10 codes 019–043), and other causes (ICD-10 code 010), as classified by the NCHS.

### Covariates

Demographic, socioeconomic, and health-related covariates were collected from household interviews and physical examinations recorded in NHANES. Age was categorized into three groups: 20–39, 40–59, and ≥60 years. Gender was classified as male or female. Race and ethnicity were categorized as Mexican American, Other Hispanic, Non-Hispanic white, Non-Hispanic Black, and Other Race. Educational attainment was grouped into less than high school, high school or equivalent, and more than high school. The family poverty income ratio (PIR) was grouped as <1.3, 1.3–3.5, and ≥3.5. The BMI was calculated as weight (kg) divided by height (m) squared and categorized as underweight (<18.5), normal (18.5–24.9), overweight (25–29.9), and obese (≥30). The smoking status was categorized as never smoker, former smoker, or current smoker. The alcohol drinking status was classified as “yes” or “no” based on self-reported behavior. Physical activity was categorized into none, moderate, and vigorous. Diabetes and hypertension were determined by self-reported diagnosis, medication use, or measured indicators, and classified as “yes” or “no.” Adjustments were made for the covariates in the analysis to control for potential confounding factors.

### Statistical analysis

All statistical analyses accounted for the complex sampling design of NHANES, incorporating sample weights, clustering, and stratification to ensure accurate estimates. Weighted adjustments were used to correct unequal selection probabilities and non-response bias. For our pooled 2005–2018 analysis, we applied adjusted examination weights (wtmec2yr = original weight/7), and specified clustering (SDMVPSU) and stratification (SDMVSTRA). Analyses were executed using survey-weighted procedures in R (survey package v4.1, developed by Thomas Lumley, University of Washington).

Continuous variables were summarized as mean ± standard deviation (SD), and categorical variables were presented as frequencies and percentages. Baseline characteristics across PINI quartiles were compared using one-way ANOVA for continuous variables and Pearson's chi-square test for categorical variables.

The associations between the PINI and mortality outcomes were evaluated using weighted Cox proportional hazards regression models. Participants were classified into quartiles (Q1–Q4) based on their PINI values. Cox models were used to estimate hazard ratios (HRs) and 95% confidence intervals (CIs) for the risk of all-cause and cause-specific mortality across PINI quartiles. Three models were developed: model 1, which was adjusted for age, gender, race, and ethnicity; model 2, which was additionally adjusted for BMI, poverty income ratio (PIR), education level, and marital status; and model 3, which was further adjusted for smoking status, alcohol consumption, and history of diabetes or hypertension. Kaplan–Meier survival curves were generated to visualize survival differences across PINI quartiles, with comparisons made using the log-rank test.

Subgroup analyses were conducted to assess the consistency of associations across different demographic and clinical subgroups, stratified by key covariates. All statistical tests were two-sided, with a *P*-value of <0.05 considered statistically significant. The statistical analysis was conducted using the statistical computing and graphics software R (version 4.2.1, developed by the R Core Team, The Comprehensive R Archive Network) and EmpowerStats (version 5.0, developed by X&Y Solutions, Inc., Boston, MA).

## Results

### Baseline characteristics

A total of 6,610 participants were deemed eligible for inclusion in the study after applying the pre-defined inclusion and exclusion criteria to the initial cohort of 70,190 participants from NHANES 2005–2018. Table 1 presents the baseline characteristics of the study participants (*N* = 6,610) stratified by PINI quartiles. The largest proportion of participants was aged between 40 and 59, and 59.55% of all participants were female. Participants in the highest PINI quartiles tended to be younger, more likely to be male, and had a lower BMI. Additionally, they had attained higher educational levels, never smoked, and were more often married or living with a partner. In the dietary intake, participants in the highest PINI quartiles tended to have higher intake of calories, protein, and dietary fiber. In contrast, those in the lowest quartile had higher rates of diabetes, hypertension and used alcohol.

**Table 1:** Characteristics of adults with depression in NHANES from 2005 to 2018.

**Characteristic**	**Total (*n =* 6,610)**	**Q1 (*n =* 1,653)**	**Q2 (*n =* 1,618)**	**Q3 (*n =* 1,591)**	**Q4 (*n =* 1,748)**	***P*-value**
**Age (years)**						<**0.001**
20–39	2,184 (33.04%)	414 (25.05%)	411 (25.40%)	542 (34.07%)	817 (46.74%)	
40–59	2,376 (35.95%)	622 (37.63%)	585 (36.16%)	577 (36.27%)	592 (33.87%)	
>=60	2,050 (31.01%)	617 (37.33%)	622 (38.44%)	472 (29.67%)	339 (19.39%)	
**Gender**						<**0.001**
Male	2,684 (40.61%)	434 (26.26%)	575 (35.54%)	689 (43.31%)	986 (56.41%)	
Female	3,926 (59.39%)	1,219 (73.74%)	1,043 (64.46%)	902 (56.69%)	762 (43.59%)	
**Race and ethnicity**						<**0.001**
Mexican American	986 (14.92%)	225 (13.61%)	229 (14.15%)	280 (17.60%)	252 (14.42%)	
Other Hispanic	721 (10.91%)	181 (10.95%)	181 (11.19%)	166 (10.43%)	193 (11.04%)	
Non-Hispanic White	2,986 (45.17%)	660 (39.93%)	728 (44.99%)	734 (46.13%)	864 (49.43%)	
Non-Hispanic Black	1,331 (20.14%)	469 (28.37%)	357 (22.06%)	270 (16.97%)	235 (13.44%)	
Other race	586 (8.87%)	118 (7.14%)	123 (7.60%)	141 (8.86%)	204 (11.67%)	
**Alcohol drinking status**						<**0.001**
No	1,805 (27.31%)	544 (32.91%)	470 (29.05%)	427 (26.84%)	364 (20.82%)	
Yes	4,805 (72.69%)	1,109 (67.09%)	1,148 (70.95%)	1,164 (73.16%)	1,384 (79.18%)	
**BMI (kg/m** ^2^ **)**						<**0.001**
Underweight (< 18.5)	127 (1.92%)	21 (1.27%)	16 (0.99%)	36 (2.26%)	54 (3.09%)	
Normal (18.5–24.9)	1,522 (23.03%)	223 (13.49%)	299 (18.48%)	389 (24.45%)	611 (34.95%)	
Overweight (25.0–29.9)	1,874 (28.35%)	365 (22.08%)	443 (27.38%)	499 (31.36%)	567 (32.44%)	
Obesity (≥30.0)	3,087 (46.70%)	1,044 (63.16%)	860 (53.15%)	667 (41.92%)	516 (29.52%)	
**Smoke status**						**0.739**
Never smoker	3,056 (46.23%)	760 (45.98%)	734 (45.36%)	760 (47.77%)	802 (45.88%)	
Former smoker	1,596 (24.15%)	412 (24.92%)	394 (24.35%)	378 (23.76%)	412 (23.57%)	
Now smoker	1,958 (29.62%)	481 (29.10%)	490 (30.28%)	453 (28.47%)	534 (30.55%)	
**Family PIR**						<**0.001**
< 1.3	2,858 (43.24%)	766 (46.34%)	683 (42.21%)	689 (43.31%)	720 (41.19%)	
1.3–3.5	2,448 (37.03%)	610 (36.90%)	634 (39.18%)	585 (36.77%)	619 (35.41%)	
>=3.5	1,304 (19.73%)	277 (16.76%)	301 (18.60%)	317 (19.92%)	409 (23.40%)	
**Marital status**						<**0.001**
Married/living with a partner	3,365 (50.91%)	807 (48.82%)	810 (50.06%)	841 (52.86%)	907 (51.89%)	
Widowed/divorced/separated	1,889 (28.58%)	553 (33.45%)	532 (32.88%)	427 (26.84%)	377 (21.57%)	
Never married	1,356 (20.51%)	293 (17.73%)	276 (17.06%)	323 (20.30%)	464 (26.54%)	
**Educational attainment**						**0.002**
< High school	1,877 (28.40%)	517 (31.28%)	462 (28.55%)	457 (28.72%)	441 (25.23%)	
High school	1,642 (24.84%)	421 (25.47%)	397 (24.54%)	396 (24.89%)	428 (24.49%)	
>High school	3,091 (46.76%)	715 (43.25%)	759 (46.91%)	738 (46.39%)	879 (50.29%)	
**Physical activity**						<**0.001**
None	4,108 (62.15%)	1,156 (69.93%)	1,067 (65.95%)	964 (60.59%)	921 (52.69%)	
Moderate	1,448 (21.91%)	329 (19.90%)	352 (21.76%)	370 (23.26%)	397 (22.71%)	
Vigorous	1,054 (15.95%)	168 (10.16%)	199 (12.30%)	257 (16.15%)	430 (24.60%)	
**Prevalent diabetes**						<**0.001**
No	5,521 (83.52%)	1,256 (75.98%)	1,302 (80.47%)	1,378 (86.61%)	1,585 (90.68%)	
Yes	1,089 (16.48%)	397 (24.02%)	316 (19.53%)	213 (13.39%)	163 (9.32%)	
**Prevalent hypertension**						<**0.001**
No	3,788 (57.31%)	806 (48.76%)	849 (52.47%)	953 (59.90%)	1,180 (67.51%)	
Yes	2,822 (42.69%)	847 (51.24%)	769 (47.53%)	638 (40.10%)	568 (32.49%)	
**Total calories**	2,026.64 ± 904.76	1,921.59 ± 841.19	1,996.98 ± 875.15	2,011.80 ± 921.41	2,166.93 ± 956.51	<0.001
**Total protein**	76.86 ± 36.88	71.60 ± 33.98	75.30 ± 35.75	77.02 ± 37.35	83.14 ± 39.16	<0.001
**Total dietary fiber**	15.52 ± 8.93	14.28 ± 8.44	15.14 ± 8.45	15.79 ± 9.08	16.80 ± 9.49	<0.001

Categorical variables were presented as N(%). Continuous variables are presented as mean ± SD. BMI, body mass index; PIR, poverty-to-income ratio.

### Relationship of the PINI with all-cause and cause-specific mortality

**Figure 2:**
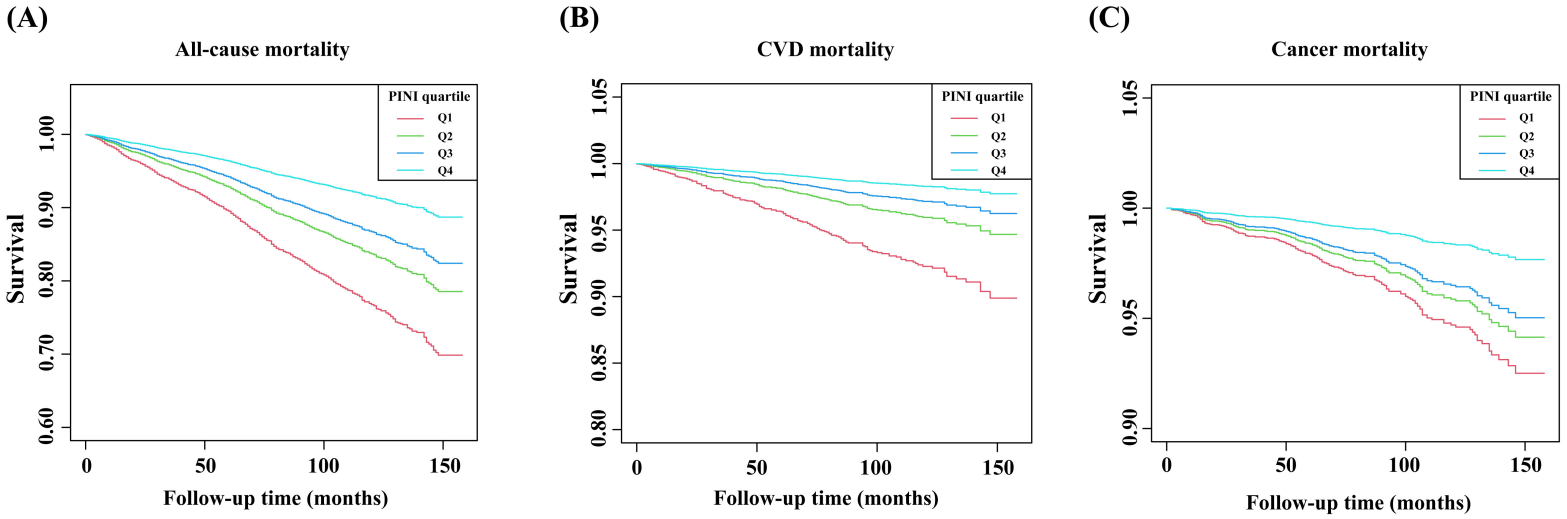
Kaplan–Meier curves of the survival rate of depressive patients with different PINI score levels. **(A)** All-cause mortality, **(B)** CVD mortality, and **(C)** cancer mortality.

Among the study participants, 702 individuals experienced all-cause mortality during the follow-up period, including 178 deaths from CVD and 153 deaths from cancer, with a median follow-up time of 6.5 years. Kaplan–Meier survival curves indicated that higher PINI quartiles were consistently associated with lower all-cause, CVD, and cancer mortality (Figure 2).

The findings suggest that using model 3, higher PINI levels were associated with decreased all-cause mortality and CVD mortality in individuals with depression. In multivariate Cox regression models, participants in the highest PINI quartile (Q4) had significantly lower adjusted hazard ratios for all-cause mortality (HR = 0.458, 95% CI: 0.349–0.603) and CVD mortality (HR = 0.258, 95% CI: 0.134–0.498), whereas the reduction in cancer mortality was less pronounced (HR = 0.554, 95% CI: 0.284–1.081) (Table 2).

**Table 2:** Associations between PINI and all-cause, CVD, and cancer mortality of participants with depression.

**Exposure**	**Model I**	**Model II**	**Model III**
**All-cause mortality (702/6,610)**			
PINI	0.258 (0.176, 0.378) <0.0001	0.257 (0.178, 0.370) <0.0001	0.303 (0.214, 0.429) <0.0001
**PINI quartile**			
Q1	Ref	Ref	Ref
Q2	0.623 (0.485, 0.802) 0.0002	0.657 (0.516, 0.837) 0.0007	0.690 (0.551, 0.863) 0.001
Q3	0.522 (0.392, 0.695) <0.0001	0.530 (0.395, 0.710) <0.0001	0.563 (0.417, 0.760) 0.0002
Q4	0.407 (0.303, 0.547) <0.0001	0.406 (0.305, 0.540) <0.0001	0.458 (0.349, 0.603) <0.0001
**CVD mortality (178/6610)**			
PINI	0.150 (0.078, 0.290) <0.0001	0.155 (0.079, 0.303) <0.0001	0.179 (0.088, 0.365) <0.0001
**PINI quartile**			
Q1	Ref	Ref	Ref
Q2	0.399 (0.262, 0.609) <0.0001	0.418 (0.271, 0.645) <0.0001	0.432 (0.273, 0.683) 0.0003
Q3	0.352 (0.199, 0.621) 0.0003	0.362 (0.208, 0.630) 0.0003	0.376 (0.203, 0.696) 0.0018
Q4	0.218 (0.121, 0.392) <0.0001	0.229 (0.121, 0.432) <0.0001	0.258 (0.134, 0.498) <0.0001
**Cancer mortality (153/6,610)**			
PINI	0.335 (0.154, 0.728) 0.006	0.342 (0.160, 0.730) 0.006	0.356 (0.166, 0.765) 0.008
**PINI quartile**			
Q1	Ref	Ref	Ref
Q2	0.651 (0.417, 1.015) 0.058	0.678 (0.428, 1.075)0.098	0.676 (0.423, 1.079) 0.101
Q3	0.567 (0.327, 0.982) 0.043	0.580 (0.335, 1.006) 0.052	0.578 (0.325, 1.027) 0.061
Q4	0.527 (0.263, 1.059) 0.072	0.538 (0.274, 1.055) 0.071	0.554 (0.284, 1.081) 0.083

Model 1: Adjusted for age, gender, and ethnicity. Model 2: Additionally adjusted for BMI, PIR, education level, and marital status. Model 3: Additionally adjusted for physical activity, total calories, total protein, total dietary fiber, smoking, alcohol drinking, and history of diabetes or hypertension. PINI, prognostic immune and nutritional index; BMI, body mass index; PIR, poverty income ratio; CI, confidence interval; CVD, cardiovascular disease; HR, hazard ratio.

### Subgroup analysis

**Figure 3:**
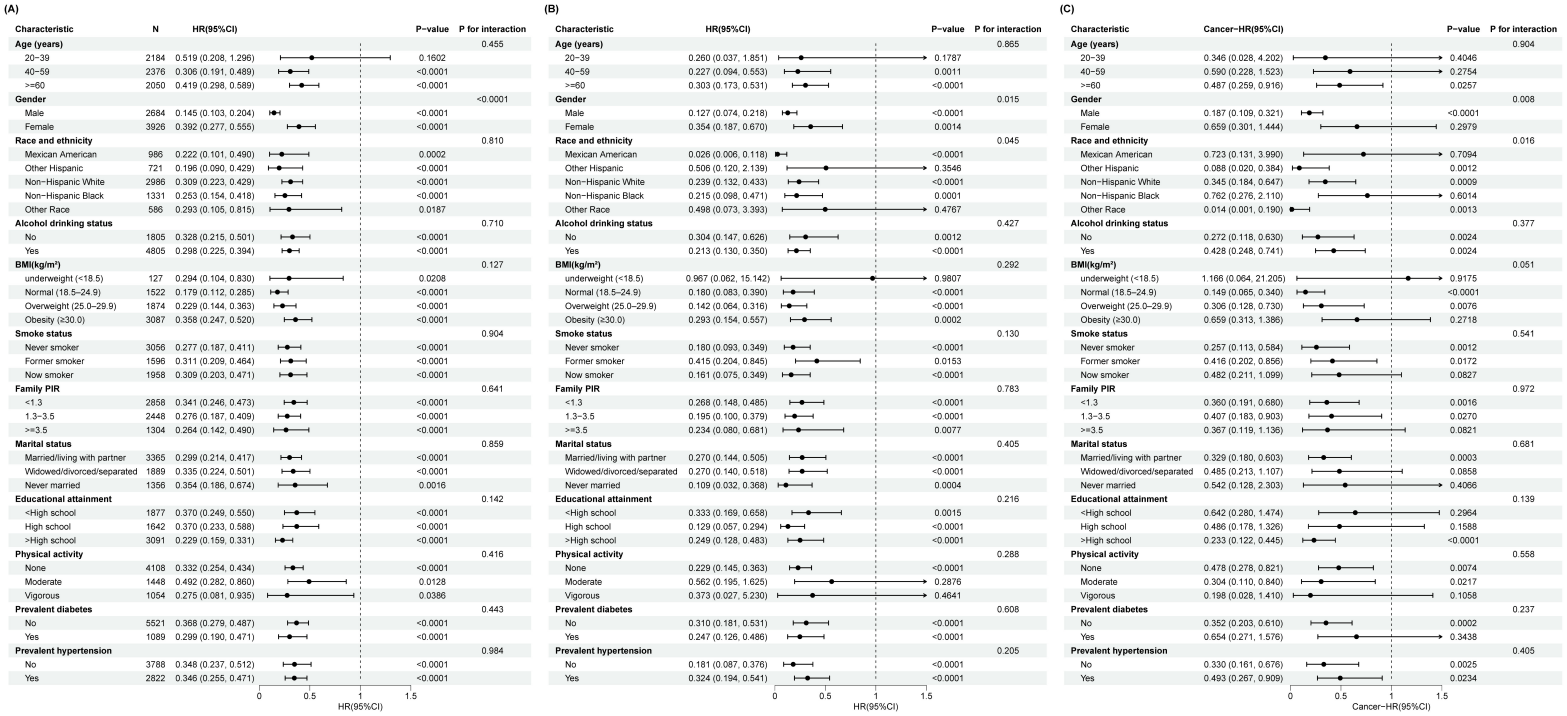
Subgroup analysis of the association between the PINI score and all-cause and cause-specific mortality. **(A)** All-cause mortality, **(B)** CVD mortality, and **(C)** cancer mortality.

As shown in Figure 3, subgroup analyses and interaction tests were conducted to investigate the relationship between the PINI and all-cause and CVD mortality based on age, sex, race, BMI, poverty income ratio, smoking status, alcohol drinking status, educational attainment, physical activity, marital status, and history of diabetes or hypertension. In Figure 3A, the HRs and 95% CIs for all-cause mortality are shown across various subgroups defined by characteristics such as age, gender, race and ethnicity, alcohol drinking status, BMI, family PIR, marital status, educational attainment, physical activity, smoking status, diabetes status, and hypertension status. The results indicate that higher PINI quartiles were generally associated with reduced risks of all-cause mortality across most subgroups.

As shown in Figure 3B, the association between the same subgroups and CVD mortality was examined. Similar to the all-cause mortality analysis, higher PINI quartiles were associated with lower risks of CVD mortality. However, the interactions observed were not as pronounced as in the all-cause mortality analysis, indicating that while the PINI may still be relevant, the nature of this relationship may be less influenced by these subgroup characteristics in the context of CVD mortality.

### The non-linear association between the PINI and mortality

**Figure 4:**
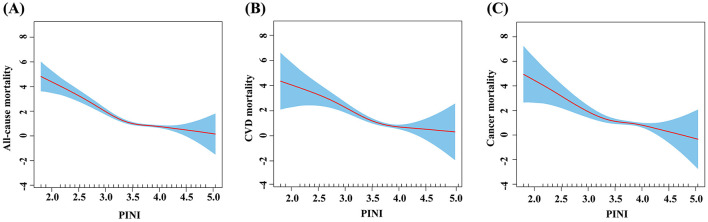
Relationship between the PINI and all-cause and cause-specific mortality in depressive patients. **(A)** All-cause mortality, **(B)** CVD mortality, and **(C)** cancer mortality. Red dots: mean; shaded area: 95% confidence interval.

By smoothed curve fitting, we discovered L-shaped associations between the PINI and all-cause (Figure 4A), CVD (Figure 4B), and cancer mortality (Figure 4C). Then, we performed a saturation effect analysis to assess the non-linear relationship between the PINI and all-cause, CVD, and cancer mortality. The results showed that there was a saturation point between the PINI and all-cause, CVD, and cancer mortality. We then explored the inflection points between the PINI and mortality. We discovered that the inflection points were 3.509 for all-cause mortality, 3.690 for CVD mortality, and 3.239 for cancer mortality (Table 3). When the PINI was below 3.509, each unit increase in the PINI was associated with an 87.1% reduction in all-cause mortality risk (0.129, 0.077–0.218, P-trend <0.0001). When the PINI was above 3.509, each unit increase in the PINI was associated with a 54.9% reduction in all-cause mortality risk (0.649, 0.375–0.804, P-trend = 0.0021). When the PINI was below 3.690, each unit increase in the PINI was associated with a 79.4% reduction in CVD mortality risk (0.206, 0.115–0.370, P-trend <0.0001). When the PINI was above 3.690, the association between the PINI and CVD mortality was not significant. When the PINI was below 3.239, each unit increase in the PINI was associated with an 81.4% reduction in cancer mortality risk (0.186, 0.056–0.622, P-trend = 0.0063). When the PINI was above 3.239, the association between the PINI and cancer mortality was not significant.

**Table 3:** Saturation effect analysis of PINI on all outcomes.

**Outcome**	**PINI**	** < K**	**>K**
All-cause mortality	3.509	0.131 (0.072, 0.239) <0.0001	0.628 (0.411, 0.961) 0.0319
CVD mortality	3.690	0.221 (0.112, 0.436) <0.0001	0.664 (0.228, 1.936) 0.4534
Cancer mortality	3.960	0.563 (0.297, 1.065) 0.0773	0.130 (0.010, 1.754) 0.1243

Age, gender, ethnicity, BMI, PIR, education level, marital status, physical activity, total calories, total protein, total dietary fiber, smoking, alcohol drinking, and history of diabetes or hypertension were adjusted. β, 95% confidence intervals (CIs), and *P*-value are presented.

## Discussion

In this study, we identified an L-shaped inverse relationship between the PINI and the risks of all-cause and CVD mortality in individuals with depression when the PINI was below certain thresholds (all-cause mortality = 3.509 and CVD mortality = 3.690). However, this relationship was attenuated for all-cause mortality when the PINI exceeded these thresholds. Our findings suggested that a better immune and nutritional status, as reflected by a higher PINI, may offer protective benefits against all-cause and cardiovascular mortality in this population. Although the association with cancer mortality was less pronounced and displayed potential age-related variations, our results highlight the prognostic value of the PINI for managing the physical health of patients with depression. Further research is needed to establish a causal relationship and validate the predictive power of our models in different populations. These results underscore the importance of integrating nutritional and immune assessments into the clinical management of depressive disorders, as poor physical health often exacerbates the adverse outcomes associated with depression.

The PINI was first developed by Jung et al. in a South Korean study that focused on the prognosis of colorectal cancer patients (16). Although some studies have suggested the PINI as an indicator of a balanced immune and nutritional status in cancer and surgical patients, further research is needed to consolidate and expand upon these initial findings. Although the condition of those with depression may not be as severe as that of cancer patients, the significant social and economic impact of depression underscores the importance of exploring effective prognostic markers in this population, such as the PINI. The observed inverse relationship between the PINI and mortality in our study builds on this body of evidence, highlighting the potential of the PINI as a valuable prognostic marker in various health contexts.

The precise reasons that the PINI is associated with mortality in patients with depressive disorders remain uncertain. However, recent studies have highlighted the potential roles of serum albumin and monocyte counts in both healthy individuals and those with depression. Serum albumin, the most abundant protein in the circulation, serves as a crucial marker of both nutritional status and the body's inflammatory response (17, 18). Its physiological functions include anti-inflammatory, antioxidant, anticoagulant, and antiplatelet activities, in addition to maintaining colloid osmotic pressure (19). Numerous studies have demonstrated an inverse relationship between serum albumin levels and the risk of dysfunction, disease onset, and mortality (20–23). In those with depression, lower serum albumin levels have been linked to higher depressive symptoms and increased mortality risk (24). For example, a study by Zhang et al. found that serum albumin levels were negatively associated with depressive symptoms in the general population (25). This suggests that albumin may act as a protective factor by reducing inflammation and oxidative stress, which are often elevated in individuals with depression.

Inflammation is increasingly recognized as a fundamental component of the pathophysiology of major depressive disorders (26). Elevated levels of inflammatory markers such as C-reactive protein and interleukin-6 have been associated with depression (10, 27), and these markers may underlie the link between the PINI and mortality outcomes. Monocytes are important cells in innate immunity and are involved in inflammatory processes. Elevated monocyte counts have been associated with increased mortality in various diseases, including cardiovascular disease and cancer (28). Elevated monocyte counts have also been observed in patients with depressive symptoms (29). For example, a study by Julla et al. found that the monocyte phenotype is a marker of cardiovascular risk in type 2 diabetes, which often co-occur with depression (30). Elevated monocyte counts may contribute to an increased mortality risk by promoting chronic inflammation, which can exacerbate the severity of depression and its associated comorbidities.

In summary, although the exact mechanisms remain to be fully elucidated, the evidence suggests that serum albumin and monocyte counts may have different effects on the relationship between the PINI and mortality in individuals with depression. Serum albumin may act as a protective factor through its anti-inflammatory activity, whereas elevated monocyte counts may contribute to increased mortality risk through their involvement in inflammatory processes. Future research should focus on further exploring these mechanisms to better understand the significance of the PINI in depression and its associated outcomes.

In the United States, 7.2% of adults have moderate-to-severe depressive symptoms, and 14.9% of them have mild symptoms. Those with mild symptoms face a 35% higher risk of all-cause mortality and a 49% higher risk of CVD mortality. For those with moderate-to-severe symptoms, the risks increases to 62% for all-cause mortality and 121% for ischemic heart disease mortality (31). The association between higher PINI levels and reduced mortality in depressed individuals may be explained by several underlying mechanisms. First, adequate nutritional intake supports immune function and reduces chronic inflammation—a known contributor to the development and progression of depression. Nutrients such as vitamins A, C, D, E, and zinc are vital for maintaining immune homeostasis and modulating inflammatory responses. Second, inflammation can lead to changes in neurotransmitter systems, including serotonin, dopamine, and glutamate, which are directly involved in regulating mood and behavior (32). For example, inflammation-induced activation of the enzyme indoleamine 2,3-dioxygenase leads to tryptophan degradation and reduced serotonin synthesis while further advancing depressive symptoms. Additionally, chronic inflammation promotes oxidative stress and neuroinflammation, further impairing neurotransmitter function and neurogenesis (32). Because higher PINI levels reflect a balanced immune and nutritional status, these processes may be mitigated, improving mental health and lowering mortality risks.

Based on the above discussion, we contend that the PINI has a critical role in the non-linear association with mortality. The results of this study indicated that when the PINI exceeded the inflection point, further increased PINI values may not indicate an increased risk of all-cause mortality but did indicate an increased risk of CVD mortality. This has particular relevance for the clinical treatment of depression. It is essential to exercise caution when treating patients with depression who have a PINI near the threshold and to re-evaluate when necessary in order to take appropriate intervention measures. In addition, subgroup analyses suggested that the association between cancer mortality and the PINI among depressive participants may be affected by gender. A substantial body of research indicates that depression is more prevalent among women than men (33–35). However, the strength of the association between depression and mortality varies considerably across studies that consider gender (36). Potential explanations for this inconsistency include sex differences in psychoendocrine, psychoimmune, and metabolic responses to depression; behavioral factors; and differences in disease detection and treatment (37). The relationship between the PINI and cancer mortality in men and women was found to be complex and requires further investigation.

However, this study has the following limitations. First, although we adjusted for various potential confounders, including demographic and health-related factors, the models with additional covariates (models 2 and 3) did not significantly alter the results compared to those of model 1. This suggested that these additional variables may have minimally impacted the observed associations. However, the potential influence of unmeasured confounders, such as genetic predispositions or environmental exposure, was not accounted for and may contribute to the observed effects. Second, the results of this study were obtained from data of depressive patients in the United States, and further investigation is essential to confirm the applicability of the conclusion to depressive populations in other countries. Cultural, dietary, and healthcare differences across various populations may influence the generalizability of our findings. For example, dietary habits and access to healthcare can vary significantly between the United States and other regions, which might influence the prevalence and impact of nutritional deficiencies and immune dysregulation on depression and mortality. Therefore, future research in diverse populations is needed to validate the applicability of our results. This highlights the importance of international studies to broaden our understanding. Third, a critical limitation of our study is that the PINI was calculated from a single blood draw. This limited our ability to assess temporal changes in the immune and nutritional status, as this is dynamic and can fluctuate over time due to various factors such as diet, stress, and illness. Single timepoint measurements may not fully capture the variability in PINI values, potentially affecting the reliability of our findings. Future research should consider serial measurements of the PINI to account for these potential temporal variations. Finally, the diagnosis of depression relied solely on the PHQ-9 scale, a self-reported measure subject to some degree of subjectivity. The PHQ-9, although widely used, is not a clinical diagnostic confirmation of depression, and therefore, its use may potentially misclassify the depression status. Therefore, individuals with mild or subthreshold depressive symptoms may have been included in the study, thereby influencing our results. Future studies should use multiple scales combined with clinician interviews to improve data reliability and diagnostic accuracy.

## Conclusion

In this study, when the PINI was below a certain threshold (all-cause mortality = 3.509 and CVD mortality = 3.690), an L-shaped inverse relationship between the PINI and the risks of all-cause and CVD mortality was observed. However, when the PINI exceeded this threshold, this relationship with all-cause mortality was attenuated. These findings suggested that a better immune and nutritional status may be protective and reduce mortality risks among depressed individuals. The role of serum albumin and monocyte counts in inflammation may partly explain this association. Integrating the PINI into clinical assessments could help identify high-risk individuals and guide appropriate interventions. Further research is needed to validate these findings across different populations and clarify the mechanisms underlying the association between the PINI and mortality in depressive patients.

## Data Availability

The raw data supporting the conclusions of this article will be made available by the authors, without undue reservation.
